# Supplementary material for: L-RNA aptamer-based CXCL12 inhibition combined with radiotherapy and bevacizumab in newly-diagnosed glioblastoma: expansion of the phase I/II GLORIA trial
Source: Nat Commun. 2026 Apr 8;17:3405. doi: 10.1038/s41467-026-71362-7 (PMC13068908; doi:10.1038/s41467-026-71362-7)
Supplement: Supplementary file 2 — Description of Additional Supplementary Files [file 41467_2026_71362_MOESM2_ESM.pdf]

Title: Supplementary Data 1
Description: Complete listing of adverse events

Title: Supplementary Data 2
Description: Complete listing of treatment-emergent adverse events per dose level.

Title: Supplementary Data 3
Description: Complete listing of treatment-emergent adverse events related to underlying disease and/or irradiation and/or NOX-A12 and/or BEV per dose level.

Title: Supplementary Data 4
Description: List of blocking component BC4 (naked oligos), antibodies, oligo sequences and multi-cycle reaction protocol for multiplexed immunofluorescence
