# Supplementary material for: L-RNA aptamer-based CXCL12 inhibition combined with radiotherapy and bevacizumab in newly-diagnosed glioblastoma: expansion of the phase I/II GLORIA trial
Source: Nat Commun. 2026 Apr 8;17:3405. doi: 10.1038/s41467-026-71362-7 (PMC13068908; doi:10.1038/s41467-026-71362-7)
Supplement: Supplementary file 7 — Reporting Summary [file 41467_2026_71362_MOESM7_ESM.pdf]

nature portfolio

Corresponding author(s): Professor Frank Giordano

Last updated by author(s): Feb 3, 2026

Reporting Summary

Nature Portfolio wishes to improve the reproducibility of the work that we publish. This form provides structure for consistency and transparency in reporting. For further information on Nature Portfolio policies, see our Editorial Policies and the Editorial Policy Checklist.

Statistics

For all statistical analyses, confirm that the following items are present in the figure legend, table legend, main text, or Methods section.

n/a	Confirmed	
☐	☒	The exact sample size (n) for each experimental group/condition, given as a discrete number and unit of measurement
☒	☐	A statement on whether measurements were taken from distinct samples or whether the same sample was measured repeatedly
☐	☒	The statistical test(s) used AND whether they are one- or two-sided<br>*Only common tests should be described solely by name; describe more complex techniques in the Methods section.*
☒	☐	A description of all covariates tested
☐	☒	A description of any assumptions or corrections, such as tests of normality and adjustment for multiple comparisons
☐	☒	A full description of the statistical parameters including central tendency (e.g. means) or other basic estimates (e.g. regression coefficient) AND variation (e.g. standard deviation) or associated estimates of uncertainty (e.g. confidence intervals)
☐	☒	For null hypothesis testing, the test statistic (e.g. F, t, r) with confidence intervals, effect sizes, degrees of freedom and P value noted<br>*Give P values as exact values whenever suitable.*
☒	☐	For Bayesian analysis, information on the choice of priors and Markov chain Monte Carlo settings
☒	☐	For hierarchical and complex designs, identification of the appropriate level for tests and full reporting of outcomes
☐	☒	Estimates of effect sizes (e.g. Cohen's d, Pearson's r), indicating how they were calculated

Our web collection on statistics for biologists contains articles on many of the points above.

Software and code

Policy information about availability of computer code

Data collection	Graphical elements were generated using R version 4.2.2, GraphPad Prism 10 (GraphPad Software) and Adobe Illustrator 2023 (Adobe Inc.). Illustrations in this manuscript were in part created using Servier Medical Art, provided by Servier, and licensed under a Creative Commons Attribution 4.0 Unported License (https://smart.servier.com). Database management (eCRF) was carried out using Viedoc version 4.66 eCRF (Viedoc Technologies) and Microsoft Excel 2019 (Microsoft Corporation). Statistical tests were performed using GraphPad Prism 10 and R version 4.2.2 as specified in the figure legends. For mIF, Imaging cycles were performed using an Akoya Phenocycler™ instrument and CODEX® instrument manager software (Akoya Biosciences). Spatial transcriptomics data were processed and visualized using Xenium Explorer (version 4.1.0, 10x Genomics).
Data analysis	Statistical tests were performed using GraphPad Prism 10 and R version 4.2.2 as specified in the figure legends. For mIF, Imaging cycles were performed using an Akoya Phenocycler™ instrument and CODEX® instrument manager software (Akoya Biosciences). Spatial transcriptomics data were processed and visualized using Xenium Explorer (version 4.1.0, 10x Genomics). The bulk tumor dataset Glioblastoma Multiforme from the TCGA Research Network was downloaded from cbioportal59 under https://www.cbioportal.org/study/summary?id=gbm_tcga_pan_can_atlas_2018. The GBM dataset with regional tissue mapping from Puchalski et al.42 was downloaded from the IVY GAP Glioblastoma Atlas Project (https://glioblastoma.alleninstitute.org/static/home). Data was analyzed and visualized using R version 4.2.2 and Graph Pad Prism 10.

For manuscripts utilizing custom algorithms or software that are central to the research but not yet described in published literature, software must be made available to editors and reviewers. We strongly encourage code deposition in a community repository (e.g. GitHub). See the Nature Portfolio guidelines for submitting code & software for further information.

Data

Policy information about availability of data

All manuscripts must include a data availability statement. This statement should provide the following information, where applicable:

- Accession codes, unique identifiers, or web links for publicly available datasets
- A description of any restrictions on data availability
- For clinical datasets or third party data, please ensure that the statement adheres to our policy

The study protocol is made available in the Supplementary Information. Source data are provided with this paper. Supplementary Figure 2 as well as high-resolution images of Supplementary Fig. 11 and Supplementary Fig. 12 are provided in the following repository: https://doi.org/ 10.5281/zenodo.18674810. Raw data from spatial transcriptomics are provided in the following repository: https://doi.org/ 10.5281/zenodo.18674925. The publicly available GBM dataset with regional tissue mapping used in this study from Puchalski et al.42 can be accessed via the IVY GAP Glioblastoma Atlas Project under https://glioblastoma.alleninstitute.org/static/ home. The publicly available bulk GBM tumor dataset from the TCGA Research Network39 can be accessed under https://www.cbioportal.org/study/summary?id=gbm_tcga_pan_can_atlas_2018. Individual participant data are not publicly available due to patient privacy regulations. De-identified data underlying the findings can be made available upon reasonable request to the corresponding authors, subject to approval by the institutional ethics committee and data transfer agreement. The remaining data are available within the Article, Supplementary Information or Source Data file.

Research involving human participants, their data, or biological material

Policy information about studies with human participants or human data. See also policy information about sex, gender (identity/presentation), and sexual orientation and race, ethnicity and racism.

Reporting on sex and gender	There were no restrictions regarding sex or gender of participating patients and male as well as female patients participated (see below). Sex was determined based on self-report. The guidance on Sex and Gender reporting was followed and corresponding statements are included in the manuscript. Given the rather low sample size reported in this study, we feel a disaggregation for sex and gender does not contribute to the conclusions of this work. However, all information on individual outcomes of patients of all sexes are provided for the interested reader.
Reporting on race, ethnicity, or other socially relevant groupings	We do not report on race, ethnicity or other relevant groupings of participants. No such restrictions applied for inclusion.
Population characteristics	All patients were first-diagnosed and neuropathologically confirmed as glioblastoma, IDH-wildtype (CNS WHO grade 4) according to the WHO classification for CNS tumors 2021 by immunohistochemistry (IHC). The median age at diagnosis of the full trial population (n = 16) was 63 (range 43 to 79) years. Of these, 14 patients had undergone partial resection; two were not amenable to resection and received biopsy only. No patient had undergone gross total resection. Eleven patients considered themselves male and five female.
Recruitment	Inclusion criteria of the dose-escalation arm of the trial were age ≥ 18 years, incompletely resected or biopsied GBM (detectable postoperative residual tumor), absence of MGMT promoter (hyper)methylation, Eastern Cooperative Oncology Group (ECOG) performance score ≤ 2, estimated life expectancy ≥ 3 months, stable or decreasing dose of corticosteroids and adequate hepatic and renal function. Inclusion criteria of the expansion arm with BEV (Arm A) additionally allowed the inclusion of patients with fully resected tumors. Sex was determined based on self-report. All patients were neuropathologically confirmed as glioblastoma, IDH-wildtype (CNS WHO grade 4) according to the WHO classification for CNS tumors 2021 by immunohistochemistry (IHC). If patients were ≤ 54 years of age, they were assessed additionally by pyrosequencing for IDH1 and IDH2. GLORIA was conducted at six academic centers in Germany, whereas the protocol was approved by ethic committees at each participating site. Each patient provided written informed consent in accordance with established guidelines. No trial participant received financial compensation.
Ethics oversight	GLORIA was conducted at six academic centers in Germany, whereas the protocol was approved by ethic committees at each participating site (ethic committees of the university hospitals of Mannheim, Bonn, Leipzig, Essen, Tübingen, and Münster). The study design and conduct complied with all relevant regulations regarding the use of human study participants. The trial followed the guidelines of the Declaration of Helsinki and the International Conference on Harmonization Good Clinical Practices Guidelines. Each patient provided written informed consent in accordance with established guidelines. The trial was reviewed by an independent data safety and monitoring committee.

Note that full information on the approval of the study protocol must also be provided in the manuscript.

Field-specific reporting

Please select the one below that is the best fit for your research. If you are not sure, read the appropriate sections before making your selection.

[x] Life sciences [ ] Behavioural & social sciences [ ] Ecological, evolutionary & environmental sciences

For a reference copy of the document with all sections, see nature.com/documents/nr-reporting-summary-flat.pdf

Life sciences study design

All studies must disclose on these points even when the disclosure is negative.

Sample size	No formal sample size calculations were performed for this standard dose-escalation trial. The dose escalation was designed as a 3+3 rule-

based design according to Le Tourneau et al. with three successional cohorts consisting of three patients each. Patients of DL 1 were to be treated with a weekly dose of 200 mg, of DL 2 with a weekly dose of 400 mg and of DL 3 with a weekly dose of 600 mg NOX-A12. After four weeks of treatment of the first patient of DL 1, the data safety monitoring board (DSMB) reviewed all DLTs, AEs, and relevant laboratory values. During the following ten weeks of treatment, the DSMB was kept informed continuously about all DLTs and SAEs, and, at the end of this period, reviewed all DLTs, AEs, and relevant laboratory values including NOX-A12 plasma concentrations prior to enrolment of the next two patients of this DL. The evaluation was repeated prior to enrolling patients in DL 2 and after patients 2 and 3 received at least four weeks of treatment. The same procedures were performed prior to enrolment of further patients in DL 2 and for DL3. If none of the three patients in any DL experienced a DLT, another three patients were to be treated at the next higher DL. However, if one of the three patients in a DL experienced a DLT, three more patients were to be treated at the same DL. The dose escalation was planned to be continued until at least two patients among a cohort of three to six patients experienced DLT (i.e., ≥ 33% of patients with a DLT at that DL), but the dose would not be escalated beyond 600 mg/week. The recommended dose for phase II trials was defined as the DL just below this toxic dose level, or 600 mg/week if this DL is not toxic. As reported, a total of 10 patients was enrolled and no DLTs were observed, while treatment with RT and NOX-A12 was safe and well tolerated. Sequentially, six additional patients were included into an expansiom arm (arm A), where patients additionally received bevacizumab in combination with the highest dose of NOX-A12 previously tested, 600 mg/week. Treatment was safe and well-tolerated in the expansion arm as well.

This non-randomized, non-blinded phase I/II study is subject to potential selection and observational biases. Participation may have favored patients with better functional status or higher motivation, introducing self-selection bias. The small sample size and lack of randomization limit representativeness and increase the risk of baseline imbalances. The absence of blinding may have influenced assessment of subjective outcomes.

Data exclusions

No data were excluded from the analysis.

Replication

Results cannot be replicated, since they represent findings in individual humans with glioblastoma that were treated with experimental or standard of care treatment.

Randomization

We report on a non-randomized multicentric phase I/II study of RT in combination with NOX-A12 and bevacizumab in first-line partially resected or unresected GBM (CNS WHO grade 4) patients with unmethylated MGMT promoter. To benchmark tissue and outcome, we established a reference cohort of GBM patients treated outside of the study with SOC RT and temozolomide (TMZ) and acquired data of additional external high-quality SOC cohorts.

Blinding

Not relevant, as no randomization took place (see above).

Reporting for specific materials, systems and methods

We require information from authors about some types of materials, experimental systems and methods used in many studies. Here, indicate whether each material, system or method listed is relevant to your study. If you are not sure if a list item applies to your research, read the appropriate section before selecting a response.

Materials & experimental systems

n/a	Involved in the study	
☐	☒	Antibodies
☒	☐	Eukaryotic cell lines
☒	☐	Palaeontology and archaeology
☒	☐	Animals and other organisms
☐	☒	Clinical data
☒	☐	Dual use research of concern
☒	☐	Plants

Methods

n/a	Involved in the study	
☒	☐	ChIP-seq
☒	☐	Flow cytometry
☐	☒	MRI-based neuroimaging

Antibodies

Antibodies used

The following antibodies were used: Ki-67, clone B56, BD Biosciences, Cat.# 556003 (RRID:AB_396287); SDF1/CXCL12, clone 79018, Thermo Fisher, Cat.# MA5-23759 (RRID:AB_2608711); α-SMA, clone 1A4, ThermoFisher, Cat.# 14-9760-82 (RRID:AB_2572996); CD31, clone EP3095, Abcam, Cat.# ab226157; GFAP, clone 2.2B10, Thermo Fisher Scientific, Cat.# 13-0300 (RRID:AB_2532994); CD68, clone KP-1, Biolegend, Cat.# 916104 (RRID:AB_2616797); CA9, polyclonal, NovusBio, Cat.# NB100-417.

Validation

All antibodies were purchased from commercial suppliers as indicated abobe and have been previously validated for the described purpose by the manufacturer or independent third parties. Validation data and applicable citations are available on product listings (see individual catalog numbers). For the reader's convenience, we added available RRID for all antibodies used (see above).

Clinical data

Policy information about clinical studies

All manuscripts should comply with the ICMJE guidelines for publication of clinical research and a completed CONSORT checklist must be included with all submissions.

Clinical trial registration

EudraCT: 2018-004064-62; ClinicalTrials.gov: NCT04121455

Study protocol

Besides the information provided in the official trial registration pages listed above, the trial protocol is provided alongside this

publication.

Data collection

Following adequate cranial wound healing and implantation of a venous port catheter, treatment with NOX-A12 was initiated within six weeks post cranial surgery. In the dose escalation cohorts, after an initial dose of 70, 160 or 230 mg per day respectively on day 1, patients were administered a fixed dose of 200, 400 or 600 mg NOX-A12 per week (DL 1, DL 2, DL 3) by continuous (24 h) i.v. infusion over a commercially-available closed pump system (CADD®-Solis VIP Ambulatory Infusion Pump by Smiths Medical) starting on day 1. Treatment with NOX-A12 ended after 26 weeks. In Expansion Arm A, patients received BEV by i.v. infusion at doses of 10 mg/kg every 2 weeks for 26 weeks. Patients with disease progression during the 26-week treatment period continued treatment with all assessments if deemed appropriate by the investigator. Continuation of treatment with NOX-A12 and, if applicable, also BEV beyond 26 weeks was allowed as per each investigator's decision, if the patient had clear clinical benefit. No simultaneous systemic oncologic treatment was permitted. Clinical and radiographic follow-up assessments included standard and advanced MRI sequences. The primary endpoint of the trial was safety as per the incidence of AEs. Secondary endpoints included NOX-A12 plasma levels, maximum tolerable dose (MTD), RP2D, imaging parameters with a specific emphasis on monitoring re-vascularization, topography of recurrence, PFS, OS and clinician/patient reported outcomes (CRO/PRO). RT was initiated on day 2 after start of NOX-A12 (and BEV) and administered as intensity-modulated, image-guided RT. Patients in the dose escalation arm received RT in a normofractionated (2 Gy per fraction) or hypofractionated (2.67 Gy per fraction) fashion up to cumulative doses of 60 or 40.05 Gy, respectively. For the expansion arm, all patients underwent normofractionated RT with 2 Gy per fraction to a cumulative dose of 60 Gy. Patients with disease progression during the 26-week treatment period continued treatment with all assessments if deemed appropriate by the investigator. Continuation of treatment with NOX-A12 beyond 26 weeks was allowed as per each investigator's decision, if the patient had clear clinical benefit. No simultaneous systemic oncologic treatment was permitted. Clinical and radiographic follow-up assessments included standard and advanced magnetic resonance imaging (MRI) sequences. The first patient was enrolled on October 07, 2019. The last patient of the dose-escalation arm was enrolled on September 2, 2021. The last patient of the expansion arm with BEV was enrolled on May 23, 2022. As further outlined in the provided study protocol, all data was collected in the participating study centers of the university hospitals of Mannheim, Bonn, Leipzig, Essen, Tübingen, and Münster.

Outcomes

The primary endpoint of the trial was safety as per the incidence of AEs. Secondary endpoints included NOX-A12 plasma levels, imaging parameters with a specific emphasis on monitoring re-vascularization, PFS, OS and clinician/patient reported outcomes (CRO/PRO).

Plants

Seed stocks

NA

Novel plant genotypes

NA

Authentication

NA

Magnetic resonance imaging

Experimental design

Design type

NA

Design specifications

NA

Behavioral performance measures

NA

Acquisition

Imaging type(s)

structural, diffusion, perfusion - no fMRI, no psychological testing

Field strength

not defined

Sequence & imaging parameters

MRI imaging sequences included: 3D T1-weighted volumetric imaging (3D T1), T2-fluid-attenuated inversion recovery (FLAIR) imaging, diffusion-weighted imaging (DWI), T1-weighted dynamic contrast-enhanced perfusion imaging (DCE), T2-weighted turbo spin-echo imaging (T2 TSE), T2-weighted dynamic susceptibility contrast-enhanced perfusion imaging (DSC), and post-contrast 3D T1 imaging. The following additional advanced imaging parameters were calculated: diffusion-weighted imaging (DWI)-derived ADC; diffusion susceptibility contrast (DSC)-derived leakage-corrected normalized rCBV and threshold-calculated FTBhigh (rCBV > 1.75); dynamic contrast-enhanced (DCE)-derived transfer constant of contrast agent (Ktrans) between the blood and the extravascular extracellular space (EES), fractional EES volume (ve), and fractional plasma volume (vp).

Area of acquisition

whole brain

Diffusion MRI ☒ Used ☐ Not used

Parameters: as per radiologist's discretion with consecutive central quality check

Preprocessing

Preprocessing software	Following acquisition, MRI images were uploaded to a secure online portal (decidemedical, Clinflows) where a central quality check was performed. All image post-processing and interpretation was performed using IB NeuroTM (Imaging Biometrics), Olea Sphere (Olea Medical) and Mint LesionTM (Mint Medical GmbH) software and assessed by a central reader not involved in the treatment of the patients (SB).
Normalization	NA
Normalization template	NA
Noise and artifact removal	NA
Volume censoring	NA

Statistical modeling & inference

Model type and settings	NA
Effect(s) tested	NA

Specify type of analysis: ☐ Whole brain ☐ ROI-based ☒ Both

Anatomical location(s): All MRI images were uploaded to an imaging database and outcome was centrally assessed by a board-certified radiologist with expertise in the field blinded for study site and clinical status. Target (TL) and non-target lesions (NTL) were identified, validated and assessed in regard to tumor size (SPD) and corresponding timepoint tumor response according to the modified Criteria for Radiographic Response (mRANO). For details, see the manuscript and trial protocol. If a patient enrolled had a singular residual tumor lesion meeting the inclusion criteria, while not qualifying for a target lesion (<10 mm in at least one diameter as per mRANO), the lesion was documented as NTL. New non-measurable contrast-enhancing lesions only constituted progression in case of complete response (CR). NTLs only impacted the response assessment in the case of a complete response of TLs. Preliminary tumor progression

Statistic type for inference (See Eklund et al. 2016)	NA
Correction	NA

Models & analysis

n/a	Involved in the study
☒	☐ Functional and/or effective connectivity
☒	☐ Graph analysis
☐	☒ Multivariate modeling or predictive analysis

Multivariate modeling and predictive analysis: Survival rates were estimated using the Kaplan-Meier method and statistically assessed by log-rank test and Cox proportional hazards regression. For Cox proportional hazards regression, a time to event variable (PFS or OS) was analyzed in regard to the event variable (censored or event) for two patient cohorts providing a hazard ratio, its 95% confidence interval and a p-value.
